# Supplementary material for: Older adults experiences of transitioning to housing following homelessness from a perspective of ontological security: A secondary analysis
Source: PLOS Ment Health. 2025 Oct 24;2(10):e0000445. doi: 10.1371/journal.pmen.0000445 (PMC12798620; doi:10.1371/journal.pmen.0000445)
Supplement: S1 Text — (DOCX) [file pmen.0000445.s001.docx]

# **Appendix A. Interview Protocol**

**Interview Protocol**

1. Tell me about your experiences of getting your own place after living in shelters/on the street. What worked well/didn’t work so well?

**Services**

1. When you were making the transition back into your own place, was there anything that you needed individually that you found you received from family/friends, service providers, or others in the community that you found especially helpful? What were they? Was there anything that you felt you needed, but didn’t receive from family/friends, service providers, or others in the community? What were they?
2. Are there services that you wish you had access to that you didn’t have when you moved into your place, or that you don’t have now? What are they?
3. Are you receiving services that are particularly helpful/unhelpful? What specifically is helpful/unhelpful about these services?

**Housing**

1. What about your housing may support or detract from your mental well-being? (e.g. people you live with, quality of your housing, neighbourhood). Do you have any suggestions for how family and friends, service providers, governments, and organizations can help those leaving homelessness to find housing that supports their mental well-being?
2. Are there times when you feel like you just want to go back to the shelter or live on the street? Why or why not?

**Community Integration**

1. Have you been able to find or keep family and friends that are good for your mental well-being? What has helped you to do this? If you wish you have more family and friends in your life that are good for your mental well-being, what are they?
2. Do you spend much time doing activities in the community outside of your place? If so, what is helping you to be involved in these activities? If not, what might help you to be more involved in these activities?
3. In what ways do you feel like you belong in your community or not? What is helping you to feel like you belong, or preventing you from feeling a sense of belonging? What could family and friends, service providers, governments or organizations do to help you to have a sense of belonging?

**Mental Well-Being and Substance Use**

1. What do you need to be mentally well and feel like you’re thriving now that you’re housed?
2. If you use drugs or alcohol, have you been able to get help with using more safely or stopping altogether? If so, is this help effective? What makes it effective/ineffective from your perspective? If not, what could family and friends, service providers, governments and organizations or others do to help you use more safely or stop using?
3. If you’ve quit using alcohol or drugs or have started to use more safely since becoming housed, what is helping you do that?

**Summary**

1. Is there anything we didn’t ask about how you could be better supported to thrive in your housing that we didn’t ask or that you thought we should mention?
