# Supplementary material for: Older adults experiences of transitioning to housing following homelessness from a perspective of ontological security: A secondary analysis
Source: PLOS Ment Health. 2025 Oct 24;2(10):e0000445. doi: 10.1371/journal.pmen.0000445 (PMC12798620; doi:10.1371/journal.pmen.0000445)
Supplement: Attachment — Submitted filename: response to reviewers.docx [file pmen.0000445.s002.docx]

Dear Reviews,

Thank you for taking the time to consider our manuscript and provide feedback. Your feedback was most helpful, and we have considered it seriously. Changes and responses to your comments can be found in the table below.

| Reviewer | Comment | Response |
| --- | --- | --- |
|  | **Overall** |  |
| 1 | Thank you for your submission related to using ontological security to understand older adults transitions out of homelessness. It was an interesting read, well structured and well aligned to the journal you have submitted to. There are some changes required before I can recommend this for publication, however, this is overall a well thought-out article which highlights an under-researched area of homelessness research. There are a number of suggested changes below, however, I feel this would still class as 'minor' revisions as the underlying justification and argument made in the article is well done. I note that the lead author is an early career researcher and would like to recognise the effort that I have no doubt went into writing this article. | Thank you for your kind words and for taking the time to review our manuscript. |
| 1 | Overall, minor points: more care to be taken in phrasing throughout - some terminology used is more emotive than balanced. There were some minor spelling/punctuation mistakes which can be resolved in proofreading. | Thank you for this suggestion. Throughout making edits I have attempted to more carefully consider wording. |
|  | **Abstract** |  |
| 1 | ‘deeply affordable’ - rephrase for clarity. | Thank you for this suggestion. I was uncertain if you meant that the sentence was unclear or that the term ‘deeply affordable’ was unclear. I have addressed both points. Firstly, I altered the flow of the sentence in the abstract (p.2). Secondly, in the policy recommendations section I expanded on the meaning of ‘Deeply affordable’. (p. 30) |
|  | **Introduction** |  |
| 1 | Trauma can be understood differently - it would be good to acknowledge differing understandings of trauma and how this concept has been applied for the purpose of this article. | Thank you for this important suggestion. I have added in a definition of individual trauma (p.4) as well as some brief discussion of the importance of a systems level perspective of trauma (pp.3-4) |
| 1 | Some discussion on housing systems and homelessness in study areas would be beneficial to help set the scene. | Thank you for this suggestion. I have added some further paragraphs discussing the connection between ontological security and homelessness, including a definition of homelessness (pp.5-6) |
| 2 | I like that you connect trauma to ontological security. But to that point, in your introduction, I would keep that as your focus. Instead of saying that OS has been applied to ageing, trauma, and housing insecurity reframe this as a few examples of trauma and OS being connected… ex: when someone has a stroke, when someone lives though a natural disaster, when someone is facing long term housing insecurity. These all seem to be instances of trauma. | Thank you for this suggestion. I think you are correct and I appreciate this feedback. I have edited this paragraph to reflect that all of these are experiences of trauma (pp.4-5) |
| 2 | Literature Review:  I am not sure why there is no literature review.  I think you could have a very short one that goes into detail about housing insecurity as trauma that then affects OS.  You could have the experiences of homelessness in here. I know there is a lot of literature on homelessness, trauma, and OS. Maybe look at Kim Skobba to help you start? And then go into the specifics you mention in your intro, that people have looked at the trauma older adults particularly experience while unhoused  And then you could talk about how transitioning to housing can be traumatizing for people exiting homelessness ending with the gap you mention in your introduction, that there is not enough literature on how this specifically impacts the growing number of older adults experiencing homelessness  One suggestion I have is to look at any work that exists on older adults living in PSH or SROs (I know there is literature on these types of housing options for older adults and how they feel within them – but there will not be a lot to your point of this being understudied at a moment when we are going to see a lot more senior homelessness…) | Thank you for this suggestion, I agree that in trying to maintain the page limit I overly condensed the introduction, specifically the literature review section. I appreciate your recommendations of research to engage with in this section. I have added two new paragraphs to delve deeper into the literature and better describe the experience of homelessness (pp.5-6) |
|  | **Methods** |  |
| 1 | I’d like more information on how participants were recruited in the primary study - how were they accessed, use of gatekeepers, etc. | I have added a short sentence to address this point (p.9), specifically locations and use of gatekeepers. I have also referred readers to the primary study in order to try and maintain the page limit. |
| 1 | States trauma could be seen as an underlying presence throughout all interviews - in what way? | I appreciate you drawing my attention to the lack of clarity of this sentence. I have removed it as I feel that upon reflection it does not add anything and the answer is within the results. |
| 2 | I would say more about secondary analysis. Maybe define it. And say why it is a good method given your circumstances. Totally get it if you were a student RA but I would just spell it out a bit more. SA is… and it was ideal for me because… in fact it gets used a lot as a method because… Also in your introductory paragraph it might be helpful for you to briefly describe the project that the data came from… just so we can follow along as your readers… why this secondary analysis of this data helped you close the gap in OS research you mention. I am assuming it was a study on older adults transitioning to housing from homelessness but spelling it out helps readers follow along seamlessly | Thank you for this suggestion, I have added a few sentences in the methodology paragraph to describe what a secondary analysis is, the focus of the primary study, and the benefits of secondary analysis as a methodology. (p. 7) |
| 2 | I really appreciate your positionality section. I found a small typo for you! “There are also commonalities that led to us…” | Thank you for catching the typo, much appreciated. |
| 2 | I would also consider putting your sample n in the methods section… I was looking for it and then found it in the findings section. | I appreciate this suggestion and have added it to the second paragraph under ‘data collection’. (p.10) |
|  | **Findings** |  |
| 1 | Justify phrasing - ‘when entire world is upended’ in relation to homelessness and transitions with reference to housing literature and ontological security in intro section to qualitative findings | This phrasing represents how overall, the findings indicated that both the process of losing housing but also gaining housing could feel like the world you have grown accustomed to has changed, including the physical spaces, routines, people you spend your time with, and beliefs about yourself and societal systems. As is common in qualitative writing, and especially thematic analysis (Braun and Clarke), the phrasing of themes and the ‘essence’ is meant to be somewhat emotive and reflect “creative and interpretive stories about the data” (p. 594, Braun and Clarke, 2019). |
| 1 | Is it reasonable to say ‘losing a sense of ontological security’ as this suggests it was there, and then it wasn’t. Ontological security can be seen as more of a continuum or process rather than something that is simply lost or found. Pg. 13 | I have reflected a lot on this point and appreciate you raising it. I agree that perhaps the term ‘losing’ does elicit a somewhat binary view. I wonder though, in how the participants described their experiences if redeveloping a strong sense of ontological security was a process that demonstrated the continuum, whereas traumatic events could lead to a rather sudden loss of ontological security in which a person abruptly felt they lacked ontological security. |
| 1 | ‘Focus was spent worrying about his health’ - phrasing. Pg. 13 | I have edited the phrasing for clarity. (p.16) |
| 1 | Smiley’s experience on Pg. 14 could be developed for clarity - unclear why not allowed to stay - had they previously been banned? | Thank you for pointing this out, I have added some further information to clarify. (pp.17-18) |
| 1 | More could be said about how derelict housing options impact ontological security to better develop argument. Pg. 15 | I have added some further information about both the definition of derelict housing, and through that, how it impacts ontological security. (pp.18-19) |
| 1 | Point to develop - social structures designed to constrain and prohibit rather than support - to add how removal of possessions impacts feelings of security and being in control of one’s own life? | I appreciate this suggestion; I added a very brief sentence to add to this point. (p.20) |
| 1 | Pg. 19 - ‘faced with the uncertainty of their lives’ - clarity - do you mean lack of control over their lives? Or do you mean they life/health? I think this can be better phrased to make this distinction clear. | Thank you for the suggestion, through the editing process I removed that paragraph and integrated these points throughout other paragraphs for clarity and to make that theme more concise. |
| 1 | ‘When the world felt like it was falling apart’ - is this something a participant said or something assumed? Can this be made clear or rephrased? As you have stated, some people are able to build feelings of ontological security in places that could be seen as inherently insecure. This suggests resilience to hardship rather than the world falling apart. | Thank you for the suggestion, through the editing process I removed that paragraph and integrated these points throughout other paragraphs for clarity and to make that theme more concise. |
| 1 | There was a general lack of citations in the results section that could have supported some of the discussion/arguments made. | I have considered this comment and while I mainly wanted to focus on the participants’ experiences there were a few spots where it felt beneficial to add citations. I also added further citations to the discussion section to better support the arguments. |
| 2 | I like seeing your sample demographic characteristics. | Thank you. |
| 2 | Now that I see 10 of your 15 interviewees were unhoused and 5 were housed, I would reframe your intro… “not enough has been written on how older adults experience the trauma of homelessness or that transition to housing…” Right now with it as “the transition to housing” only I assumed your 15 were all now housed… | Thank you for this suggestion. I have edited the introduction paragraphs to better support the premise that the transition to housing is not only about what occurs following finding housing but also about the search for housing. (p. 3) |
| 2 | At the top of qualitative findings, I think you mean to say (first sentence under Essence) “the essence of the experience of transitioning from housed to unhoused… and then back to housing…” Or maybe you want to start with “going from housed to unhoused” means you learn to live in a new reality… and this is true to even as people rehouse… | I have considered your point and edited this section to reflect your second option - going from housed to unhoused” means you learn to live in a new reality… and this is true to even as people rehouse”. Thank you for the suggestion. (p. 14) |
| 2 | When you go into ageing and physical distress I would connect this more clearly to being unhoused… this sounds like something we all may go through when we age… feeling a loss of OS as we worry more about our health, but this is compounded when you are unhoused… and then of course you rightfully mention your preoccupation with health can take up all your time and make it hard to find housing… | You raise a good point; I have added some elements to more clearly explain how this is connected to being unhoused. (p.26) |
| 2 | Did Lola find housing? It is not clear. | She did not find housing at the time of the study. I have extensively edited this paragraph, and I believe it helps clarify this point. (p.16) |
| 2 | I think the one thing missing from Bambi’s story is if the shelter makes her feel less OS… this would keep your through line of homelessness and trauma | Thank you for this suggestion. For Bambi, I believe what impacted her sense of ontological security was her changing health and the difficulty of finding housing, rather than living unhoused. |
| 2 | I really like the distinction you make between people who have been unhoused for a while and have “aged on the street” so to speak versus those who have just become unhoused as seniors… both are growing trends… and both have their own traumas… | Thank you, I was very struck by this distinction when engaging with the participants interviews and the surrounding literature. |
| 2 | When you get to the part about systems being broken and specifically people feeling that they weren’t having their needs met in health settings, I would more solidly connect this to the experience of homelessness… why is it that unhoused individuals are having these poor interactions with psychiatrists? | I appreciate this suggestion. I have added some information on discrimination and stigma to help connect this to the experience of homelessness. (p.19) |
| 2 | Why did Gabriella feel overwhelmed in her new housing? | I have added a brief sentence to explain. (p.23) |
| 2 | In your learning to adapt to a new reality section it currently feels like you are doing too many things… 1) you are talking about how some people could build OS in new housing settings and how others could not on account of things like the presence of social support… but then you also discuss how people on the streets built OS together through community and are now loth to enter housing… but then you also go into what people do when they can’t improve their OS in terms of coping mechanisms… I would reorganize this section… What is it that your interviews show you? Some people redevelop OS when they move into housing because there are social supports? Others don’t because there are no social supports? By contrast those who are not connecting to housing at least have each other (i.e. social supports/community) and that gives them a new sense of OS even if it makes it harder for them to move into housing (because they don’t want to leave their friends behind? Which I have seen!)… this all to me demonstrates points of action we could take… all of which revolve around social ties…we could build up social support in PSH… move entire encampments into PSH together… maybe you want to rework this last section of your findings to focus on the through line… the importance of social supports for rebuilding OS… and maybe wait on the other stuff about acceptance and put it in your discussion… | Thank you for this comment. I have considered your points and edited this section in response. Firstly, I changed the theme name to reflect the common element of social connection. Secondly, I edited the first paragraph to reflect this focus and actually did not need to edit many other paragraphs as they already focused on social support. Finally, I removed the final paragraph and integrated these points throughout the above themes which both helped provide additional clarity to some of the above sections and made this theme more succinct. (p.22) |
|  | **Discussion** |  |
| 1 | Recommendations could be more carefully phrased - overly emotive language in places that is not always supported by the discussion above. Some of the recommendations are not discussed in the results or discussion - e.g. wish to move to supported accommodation. Rather than introduce new ideas in the recommendations, these should be in the discussion and results too or not part of the recommendations. | I reflected on this point and have extensively edited the first paragraphs of the discussion. I also returned to the results section to ensure all points mentioned in the discussion came from something I had included in the results. (pp.25-31) |
| 2 | I think you need to go back through your findings section and discussion section together and really think about what it is you found and are thus arguing. It is just a little disjointed at the moment. And when you do this I think you also need to keep the thread of “older adults” in mind… why is this specifically important for them (declining health and ability… already traumatic possibly but worse when unhoused… declining social networks with people in their lives dying/divorces…this then makes your findings more interesting for older adults… ex: why social supports may be so important for rebuilding OS either in housing or on the streets…ex: why having the rug pulled out from under you is especially scary at this age, when you have health conditions and lost abilities… ) | Thank you for this suggestion. I have gone through and extensively edited the discussion sections to make them more congruent with the results sections and more focused on the specific needs of older adults. (pp.25-31) |
