# Supplementary material for: Older adults experiences of transitioning to housing following homelessness from a perspective of ontological security: A secondary analysis
Source: PLOS Ment Health. 2025 Oct 24;2(10):e0000445. doi: 10.1371/journal.pmen.0000445 (PMC12798620; doi:10.1371/journal.pmen.0000445)
Supplement: Attachment — Submitted filename: response to reviewers August 2025.docx [file pmen.0000445.s003.docx]

Dear Reviewer,

Thank you for taking the time to consider our manuscript and provide such thoughtful feedback. I truly appreciated reading your thoughts and suggestions and believe your recommendations have strengthened the manuscript. Changes and responses to your comments can be found in the table below.

| Comment | Response |
| --- | --- |
| **Introduction and Lit review** |  |
| I really like your reworking of your introduction. I enjoy the way you frame trauma as structural in origin but deeply personal in how it is experienced, and how you lay out the myriad consequences of trauma for individuals. This lays the foundation for your topic - homelessness as structural trauma but also housing as a mediating factor... poor housing options are still structural trauma... thank you for also taking your range of topics related to ontological security (aging, homelessness, etc.) and just referring to how they all can produce trauma. I think it streamlines your paper. Thank you also for adding in a bit more about how homelessness affects OS (Skobba etc). | Thank you for this feedback. I am glad the changes made have improved the introduction. |
| My one suggestion here is to divide up your intro and lit sections. They are now one 4 page section. Having a separate intro that lays out your framework concisely and a roadmap for the rest of your paper could help readers better follow your arguments. And then you could have a separate lit review where you dive deeper into how trauma is structural in origin, how it affects individuals in multiple ways, why it is related to ontological security... and then how trauma and ontological security affect older adults experiencing homelessness. | Thank you for this suggestion. Due to page limitations, I do not want to add any further sections. However, I agree with your point that a 4-page section is slightly long and have therefore added two headings to enhance clarity. |
| If you find this feedback useful, you could also have a very abridged definition of ontological security in your intro so people know right away what it is - you can use this one you wrote - Ontological security is the sense that the world and the way that it is constructed in one’s personal life can be depended upon to remain constant (29,30). And then you can expand on what it means (the stuff you write about Giddens for example) in the lit review. Just a thought. I see though I made the same comment last time - so maybe this is a style discrepancy we have across our disciplines. | Thank you, I appreciate the suggestion. I have not created a lit review section per se. However, I did add this definition to the intro for clarity. (see pg. 3) |
| One final thing: the way you so clearly define trauma now as structural in origin it still lends itself to you thinking about aging, natural disasters, and housing insecurity as instances of trauma that affect OS but maybe you want to be clear that systems of racism, classism, etc. are what put certain people at greater risk of natural disaster or having a stroke. | Thank you for this suggestion, I have added two sentences to address this. (see pg. 5-6) |
| **Methods** |  |
| Thank you for defining secondary analysis and giving context - benefits of SA, challenges of SA, and why you could evade those challenges given your particular circumstances. | Thank you. |
| Thank you for clarifying what the original study was. It just helps readers follow along in terms of why SA was such a good fit here. | Thank you, I agree it was a beneficial change. |
| Thank you for making your sampling strategy for SA easier to find by moving it to methods. | Thank you. |
| The last two sentences of your methods section are a little hard to read. Consider revising for clarity. ‘Following the guidance of (54), a central essence regarding how older adults experience trauma cross the transition to housing following homelessness was created that connected all the themes. The final stage of the analysis was the writing, where we described what each theme is about, using participant quotes to center the participants’ experiences.’ | I appreciate this feedback. Returning to that paragraph I realized I had started writing the analysis as stages and then did not continue that style of writing. I have made minor edits to this paragraph and those two sentences for clarity. (see pg. 11) |
| **Findings** |  |
| Thank you for now being clear in your intro section and elsewhere that these are folks transitioning from homelessness to housing. This way it encompasses all your interviewees, including those still unhoused. | Thank you, I agree this has added clarity and better encompasses all participants. |
| I can't remember from the first review, but this works: mentioning your sample came from an original sample and that it is n=15 in the methods section but then going into demographic info for these 15 in the findings. | Thank you. |
| How does Lola's focus on living life in the moment make it hard for her to find housing? | I have added to the sentence to clarify. Thank you for the question. (see pg. 16) |
| Reviewing your findings again, I think you want to take another look at your framework... I am getting this picture as I read: homelessness and even the transition out of homelessness impacts OS as homelessness is traumatizing and so too is looking over subpar housing options where you feel unsafe... and what is more, when you are homeless and dealing with health issues or childhood trauma that led to homelessness these things not only erode OS but make it hard to get into housing. What helps? When people fight back against violent structures, when people have social support, and when people have adequate housing options available to them. I don't think it is wrong to have all this - I would just have you review your framework. Right now your research question is: How do older adults experience trauma across the transition to housing following homelessness? But maybe it is "how are older adults affected by trauma and what protective factors exist for them?" | Thank you for this feedback. After thinking about it extensively I believe I see your point. I have chosen to leave it as is for the following reasons. Firstly, I believe the focus on the transition is integral to the question. Secondly, I believe that the term ‘experience’ leaves space for protective factors, as having positive relationships and social support were elements that impacted the experience of trauma, and were therefore part of the experience. |
| Maybe I am thinking too deeply about this, but in your theme 2, "the system is not going to save me," this comes across for me in two ways 1) the system isn't ending my homelessness and 2) the system - ie multiple systems that interact with the unhoused, healthcare and homelessness services here specifically, is not just not ending homelessness but furthering suffering through stigmatization. It's important that your interviewees mention stigma at hospitals and at shelters. And then you end on a note where stigma is not happening, where people are treated with dignity which is so important for OS (a sense of stability in your life, a sense of stability in your identity - that these people don't see me as homeless but as a whole person). But I think maybe because you are focused on "the system" here you might want to nix the example of servers treating someone nice and focus on moments in "the system" where providers are compassionate. And on this note, maybe it would help readers follow your argument across all three themes if you clearly connect your findings to OS... like the above example I gave (when you are treated with dignity, you can maintain your sense of self...). Or how theme 1 "having the rug come out from other you" throws your sense of constancy in your life/constancy in your self out the window... | Thank you for this suggestion. I have gone through the paragraphs of this theme and centered them more carefully around the system, moving examples that did not relate to the system. (see pg. 21-22). |
| For your third theme, I have a similar comment. I would take the time to analyze each example you share. Ex: the two roommates who are both traumatized... why is it that this social tie actually works against OS... this is not a lot of work, just a sentence after each example. Does this person feel unsafe because of their roommate? Explain! It just is that much effort. Like a sentence. | I appreciate this feedback and have done as you suggested. (see pg 24). |
| And I love the finding you have that for some unsheltered individuals they build new OS together on the streets. But this makes it hard to move into housing. I think you pared that section down a bit too much. I would put that explanation of your examples back in. | Thank you for this feedback. I agree I may have overly pared down this section, I have added some further explanations following each example. (see pg. 25). |
| I also think it was smart to take out the coping part in theme 3. Your through line is the social ties - whether they are present or not/helpful or not for maintaining housing and how they are protective on the streets but prevent housing. | Thank you. I agree and thank you again for your previous suggestions that led to this change. |
| **Summary of Recommendations** |  |
| In conclusion, I don't know if you need to make a lit review. This might be a style discrepancy we are butting up against. But I do recommend putting in a line about how aging, natural disasters, and housing are all examples of structural trauma. Feel free to keep or exclude my recommendation to rework your last two sentences in Methods. I think the through line in my comments about your Findings section is just to add a bit of explanation after your examples. Be explicit. Why is this example an example of eroding OS or an example of it being preserved? I would keep this really short. Just a comb through and adding the sentences of explanation where you need them. I really hope this helps! | Thank you again for all your feedback and recommendations. I have worked to incorporate your suggestions throughout and I believe it has enhanced the manuscript. |
